# Evaluating Liquid Biopsy for Circulating Tumor DNA (ctDNA) Detection as a Complementary Diagnostic Tool in Thyroid Cancer Among Ecuadorian Women

**DOI:** 10.3390/ijms26146987

**Published:** 2025-07-21

**Authors:** Santiago Cadena-Ullauri, Viviana A. Ruiz-Pozo, Elius Paz-Cruz, Rafael Tamayo-Trujillo, Patricia Guevara-Ramírez, Oscar Jaramillo-Calvas, Cristhian García, Mikaela García, Ana Pérez, Maritza Ochoa-Castro, Fausto Zaruma-Torres, Favian Bayas-Morejón, Lenín Guamán-Herrera, Ana Karina Zambrano

**Affiliations:** 1 Centro de Investigación Genética y Genómica, Facultad de Ciencias de la Salud Eugenio Espejo, Universidad UTE, Quito 170129, Ecuador; santiagoa.cadena@ute.edu.ec (S.C.-U.);; 2 Unidad de Cirugía de Cabeza y Cuello del Hospital Carlos Andrade Marín, Quito 170402, Ecuador; 3 Instituto de la Tiroides y Enfermedades de Cabeza y Cuello (ITECC), Department of Head and Neck Surgery, Quito 170102, Ecuador; 4 Grupo PharmaGenTox del Departamento de Biociencias, Universidad de Cuenca, Cuenca 010107, Ecuador; 5 Centro de Investigación CIMABiF, Facultad de Ciencias Agropecuarias, Recursos Naturales y del Ambiente, Universidad Estatal de Bolívar, Guaranda 020150, Ecuador

**Keywords:** liquid biopsy, ctDNA, thyroid cancer, next-generation sequencing, concordance

## Abstract

Thyroid cancer (TC) is the most common endocrine malignancy, with a rising global incidence. In Ecuador, TC rates are among the highest worldwide. Generally, fine-needle aspiration (FNA) remains the standard diagnostic tool; however, due to its limitations, alternative or complementary approaches are required. In this context, liquid biopsy, particularly circulating tumor DNA (ctDNA), offers a promising, minimally invasive option for tumor genotyping. Objective: This study evaluated the concordance between genetic variants identified in ctDNA and tumor tissue. Thirty-six women with papillary thyroid cancer were included. Tumor tissue and blood samples were collected, and DNA was extracted. Next-Generation Sequencing (NGS) using the TruSight Tumor 15 panel identified genetic variants in both ctDNA and tumor DNA. Variant pathogenicity was assessed following ACMG guidelines. Genetic ancestry was determined using Ancestry Informative Markers (AIMs). A total of 71 cancer-associated variants were detected, with 81.69% concordance between tumor DNA and ctDNA. *TP53* was the most frequently mutated gene. While most pathogenic variants were found in tumor tissue, some variants appeared exclusively in ctDNA samples on specific patients, suggesting tumor heterogeneity. Ancestry analysis revealed a predominant Native American component (62.4%). Liquid biopsy demonstrates high concordance with tumor tissue analysis and holds potential as a complementary diagnostic tool for thyroid cancer. However, challenges such as low ctDNA yield and underrepresentation in genetic databases highlight the need for improved protocols and increased inclusion of admixed populations in genomic studies.

## 1. Introduction

Thyroid cancer (TC) is the most endocrine neoplasia and ranks as the tenth most common cancer worldwide [1]. The situation in Ecuador is no different, as the country exhibits one of the highest incidence rates of TC internationally [1,2,3]. TC accounts for approximately 5% of all cancer diagnoses globally and has a 5-year survival rate of 98.5% [1,4]. Concerningly, TC incidence continues to increase annually, with global TC cases projected to increase by 44.1% between 2019 and 2030 [1,2].

Clinically, TC is characterized by the formation of thyroid nodules and is classified according to the affected cell type. Tumors originating from follicular cells include papillary, follicular, and oncocytic carcinomas, while those arising from parafollicular C cells are classified as medullary thyroid carcinomas [5]. Among these, papillary thyroid cancer (PTC) is the most common subtype and is generally associated with a favorable prognosis [3].

Various risk factors have been associated with TC, including age, radiation exposure, sex, iodine intake, and ethnicity [6]. Notably, TC occurs more frequently in women across diverse populations and geographical regions [7,8]. Research has suggested a potential correlation between TC and estrogen signaling, including interactions with estrogen receptors [8]. Estrogen can have growth-promoting effects through the activation of signaling cascades such as the MAPK and PI3K pathways. In PTC, these pathways may be overactivated as a consequence of chromosomal rearrangements or mutations in the *BRAF* gene [9]. However, there are considerable inconsistencies in the literature; therefore, further research is required to elucidate the intricate interaction between these factors and TC tumorigenesis.

The primary diagnostic method is through a fine-needle aspiration (FNA) biopsy, which relies on the cytopathological assessment of smears from thyroid nodules. Despite its widespread use, FNA has various limitations, including procedural risks such as tracheal puncture, hemorrhage, and patient discomfort [10]. Furthermore, the results obtained from the FNA biopsy are not always conclusive and can be indeterminate, leading to the need to repeat the procedure, increasing its intrinsic risks [10]. Additionally, due to the high intratumoral heterogeneity, single-site tissue biopsies may not fully capture the genetic landscape of the whole tumor [10].

In this context, liquid biopsy has emerged as a promising, minimally invasive approach. This method enables the detection of multiple analytes, including circulating tumor DNA (ctDNA). ctDNA comprises cell-free fragments shed into the bloodstream by tumor cells. Interestingly, ctDNA concentration have been correlated with tumor burden, staging, and prognosis [11]. Furthermore, by coupling ctDNA and Next-Generation Sequencing (NGS), it is possible to identify somatic variants with pathogenic significance, as well as facilitating the evaluation and genotypic classification of the tumor, offering an alternative for TC diagnosis, and leading to the identification of potential TC biomarkers [12,13,14,15,16].

## 2. Results

### 2.1. Participants’ Demographics

A total of 36 Ecuadorian women diagnosed with PTC were included. The mean age of the participants was 52 years (SD: 12.34), ranging from 32 to 84 years. All the subjects underwent a partial or total thyroidectomy as part of their clinical management.

### 2.2. Genetic Mutations in Thyroid Cancer Patients

The study identified 7584 genetic variants among the genes included in the TruSight Tumor 15 panel, from both tumor tissue and ctDNA. A detailed description of each variant is provided in Supplementary Table S1. The gene with the highest number of identified variants was *TP53*.

### 2.3. Concordance Between Variants Identified

Out of the 7584 genetic variants identified, 71 cancer-associated variants identified remained after assessment. In total, 58 (81.69%) genetic variants were concordantly detected in both tumor tissue and ctDNA from 27 patients, indicating a high level of agreement between paired samples. In contrast, 11 variants (15.49%) were not concordant and were only found in tumor tissue, while 2 variants (2.82%) were detected solely in ctDNA from specific subjects. Notably, the two variants observed solely in ctDNA were also detected in the tumor tissue of different individuals within the cohort. This observation suggests potential interindividual variation in ctDNA release or detection sensitivity and highlights the complexity of interpreting variant exclusivity in liquid biopsy contexts.

### 2.4. Pathogenicity Classification

**Figure 1:**
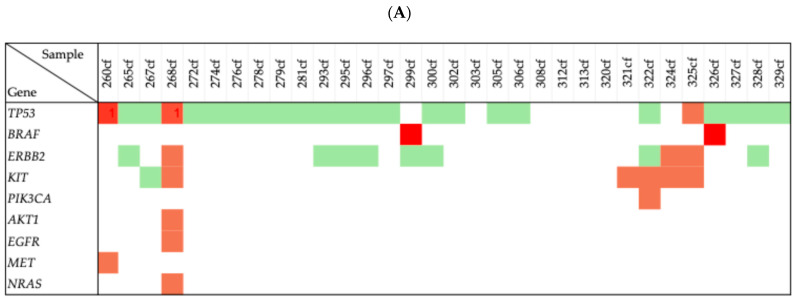

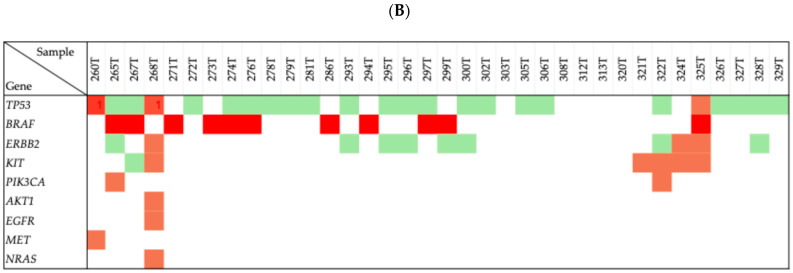
(**A**) Distribution and pathogenicity of genetic variants identified in ctDNA, and (**B**) tumor tissue. Each row represents an individual sample, and each column corresponds to a gene carrying a variant of interest. The color-coding shows the potential pathogenicity of the variants in the gene: green denotes benign or likely benign variants, orange clay denotes variants of uncertain significance (VUSs) and red indicates pathogenic or likely pathogenic variants in each gene.

Based on the guidelines of the ACMG, the identified variants were classified as follows: Benign, 30 variants, mainly based on population frequency data. Variants of Uncertain Significance (VUSs), 25 variants, classified according to population frequency and functional data, as well as in silico prediction models. Likely pathogenic, four variants, supported by population frequency data, functional studies, in silico predictions, and protein-level effects. Pathogenic, two variants, identified in both tumor tissue and ctDNA, were classified based on case–control studies, functional and population frequency data, effect on protein, and in silico predictions. For one variant, the pathogenicity could not be determined due to insufficient available evidence. Figure 1 shows the distribution, overlap and diversity of genetic variants identified in tumor tissue and ctDNA across the genes in the patient’s cohort.

### 2.5. p.Leu265Met Determination

**Figure 2:**
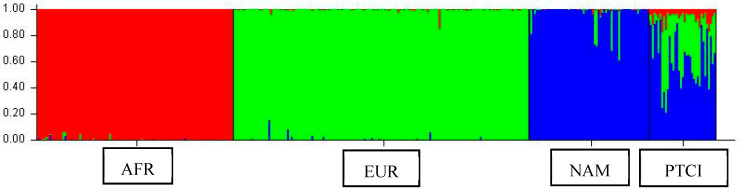
Ancestry proportion determination. The red, green, and blue sections represent the African (AFR), European (EUR), and Native American (NAM) reference populations, respectively. The sample from Papillary Thyroid Cancer individuals (PTCI) is shown at the right part of the graph.

The genetic ancestry determination revealed a predominant Native American (NAM) component (62.4%), followed by European (EUR) contribution (31.3%), and a minor African (AFR) proportion (6.3%). The ancestry proportions are depicted in Figure 2. In the graph, the higher proportion of blue represents the predominant Native American component identified in the cohort.

## 3. Discussion

Currently, no non-invasive method exists for a definitive diagnosis of thyroid carcinoma, underscoring the growing need for alternative diagnostic tools as global incidence rates continue to rise [3]. ctDNA presents a promising non-invasive option, as it originates from apoptotic and necrotic tumor cells and can reveal tumor-specific genetic alterations, thereby aiding in diagnosis and precision medicine while mitigating the risks associated with FNA [17].

Thyroid carcinoma common genetic alterations include *CTNNB1*, *BRAF*, *EGFR*, *FOXL2*, *GNAS*, *KRAS*, *NRAS*, *HRAS*, *RET-PTC3*, *TERT*, *RET-PTC1*, *PAX8-PPARɤ*, *PIK3CA*, and *TP53* mutations [18]. In our study, we identified mutations in nine genes: *AKT1*, *BRAF*, *EGFR*, *ERBB2*, *KIT*, *MET*, *NRAS*, *PIK3CA*, and *TP53*.

The *BRAF*^V600E^ mutation is the most prevalent genetic alteration observed in PTC [2]. In our study, the mutation was identified in twelve PTC patients, further supporting its strong association with the disease. Among these cases, ten mutations were exclusively detected in tissue samples, one was concurrently present in both tissue and ctDNA from the same patient, and one was found solely in ctDNA. Reported prevalence rates of the *BRAF*^V600E^ mutation in ctDNA from patients harboring the mutation at the somatic level vary widely across different populations, ranging from 0% to 91.7% [19]. For instance, studies by Cao et al. (Chinese population), Condello et al. (Italian population), and Kwak et al. (Korean population), employing NGS, qPCR (quantitative PCR), and digital PCR, detected the mutation only in tissue samples, with no corresponding detection in ctDNA [17,20,21]. In contrast, Jensen et al. (U.S. population) and Sato et al. (Japanese population), using digital PCR, reported *BRAF*^V600E^ mutation prevalences of 42.1% and 31%, respectively, in the ctDNA of patients with primary thyroid tumors [22,23]. Furthermore, Pupilli et al. (Italian population) and Patel et al. (English population) identified *BRAF*^V600E^ mutations in ctDNA in 30% and 40% of cases, respectively, who were negative for the mutation in tissue samples [14,24]. These findings highlight the variability in ctDNA detectability across populations and methodological differences, underscoring both the potential and current limitations of ctDNA analysis in the molecular characterization of PTC.

The absence of the *BRAF*^V600E^ mutation in ctDNA, observed in our study, may be attributed to several factors, including limited sample size, inter-individual variability among PTC patients, and differences in pre-analytical procedures and detection methodologies [19]. Nevertheless, our findings are consistent with previous studies that also failed to detect the mutation in ctDNA, even in patients with metastatic disease. This may be due to the typically small tumor burden and low levels of ctDNA shedding characteristic of PTC [17]. Conversely, the detection of the *BRAF*^V600E^ mutation exclusively in ctDNA may be explained by the fact that *BRAF* mutations, when they are not germline, rarely occur as clonal events [19]. A clonal event refers to a genetic mutation that is present in all tumor cells, typically arising early during tumorigenesis. In contrast, subclonal mutations arise later during tumor evolution and are present only in a subset of tumor cells [25]. Consequently, somatic testing may miss these mutations if performed on tumor regions lacking the alteration, leading to false-negative result [26]. ctDNA analysis could help overcome this limitation by capturing the genetic heterogeneity of the tumor, enhancing the sensitivity and clinical relevance of molecular diagnostics. Alternatively, this event may also be explained by the presence of other types of cancer tumors, such as melanoma or colorectal cancer, carrying the *BRAF*^V600E^ mutation and releasing its ctDNA [27].

*TP53* was the most frequently altered gene in our cohort, with twenty-four patients harboring at least one mutation, detected in both tissue samples and cfDNA. Notably, all mutations were concordant across paired samples. Of these, four mutations (p.Leu265Met, p.Asp259Ala, p.Ala74GlyfsTer75, and p.Ala74GlyfsTer50) were classified as likely pathogenic. One mutation (p.Gly361Arg) was categorized as a VUS, while the remaining mutations were classified as benign.

This may be explained by the fact that *TP53* mutations are more commonly associated with high-grade thyroid cancers, such as anaplastic thyroid carcinoma (ATC). A meta-analysis by Lacka et al. demonstrated that the frequency of *TP53* mutations was significantly higher in patients with ATC compared to controls. Furthermore, no significant difference was observed between patients with PTC and the control group [28]. Therefore, a higher frequency of mutations in *TP53* may be associated with an increased likelihood of identifying variants that could be characterized as pathogenic, as a greater number of mutational events raises the probability of affecting functionally critical regions of the gene.

In contrast, the identification of a variant classified as benign through genetic testing indicates that it is not associated with a clinically meaningful increase in disease risk. Accurate interpretation and classification of these variants are essential to avoid misinterpretations and prevent unnecessary medical interventions, such as prophylactic surgery or inappropriate treatments [29]. Although classified as benign, these variants can influence cancer biology through minimal alterations in protein function or interactions with other genetic factors [30,31].

Interestingly, we also detected a *TP53* p.Pro72Arg variant classified as benign in this study. This variant can lead to two isoforms of the P53 protein: P72 (Proline) and R72 (Arginine). De Souza et al. has revealed that the P72 variant is involved in cell cycle regulation processes, enhanced immune response, and increased activation of genes related to the P53 pathway, whereas the R72 variant is involved in apoptosis. These results suggest that individuals carrying the P72 variant may have a more favorable biological response to cancer and improved overall survival compared to those with the R72 variant [32]. These findings may contribute valuable information to understanding cancer development and progression in admixed populations.

Similarly, other benign variants such as *ERBB2* p.Ile655Val, and *KIT* p.Met541Leu were identified in the present study, suggesting no pathogenic effect despite being located in cancer-related genes. However, even benign variants could influence cancer pathogenesis by modulating genetic background and molecular mechanisms [33], reinforcing the need for their comprehensive characterization in oncology.

The classification of these VUSs remains challenging, especially in underrepresented populations such as Latin Americans, where genomic data are scarce in global databases like ClinVar and gnomAD. This underrepresentation leads to a higher prevalence of VUSs and complicates variant interpretation [34]. Therefore, accurate clinical interpretation is crucial for the eventual reclassification of s as either benign or pathogenic [35,36].

In this context, several VUSs were also detected in genes implicated in cancer-related mechanisms, including *PIK3CA*, *AKT1*, *ERBB2*, *KIT*, *EGFR*, *NRAS*, and *TP53*. For example, mutations in *PIK3CA* have been more frequently associated with anaplastic and follicular thyroid cancers and to a lesser extent with PTC [37]. *PIK3CA* is an oncogene whose aberrant expression can lead to increased phosphorylation of AKT and subsequent activation of the PI3K/Akt/mTOR signaling pathway, which promotes cancer cell survival and proliferation [38,39,40]. Similarly, variants in *ERBB2* have shown clinical relevance in various neoplasms, including advanced or metastatic PTC with poor prognosis. A study by Jin Y. et al. (2022) showed significantly higher *ERBB2* expression in PTC tissues compared to normal thyroid tissue. Furthermore, overexpression of the *ERBB2* gene is involved in processes such as the dysregulation of iodine metabolism, tumor proliferation, metastasis, and drug resistance [41].

A recurrent VUS in *KIT*, particularly p.Ala482Pro, was also observed. *KIT* is a proto-oncogene that encodes a tyrosine kinase receptor that is activated by binding to its ligand stem cell factor (SCF) and modulates different metabolic pathways such as MAPK, PI3K, and STAT, which are involved in cell proliferation and survival [42]. Tomei et al. (2012) [43] mentioned that *KIT* overexpression has been linked to tumorigenesis in several types of cancer, including hematological cancer, colon cancer, and neuroblastoma, among others. In contrast, in the case of PTC, cell dedifferentiation and tumor progression were associated with loss of *KIT* gene expression, which could be a diagnostic tool for identifying malignant thyroid nodules [2,43]. These findings align with a previously reported case in an Ecuadorian patient with PTC, in which in silico analyses of tumor tissue identified potentially pathogenic variants in *KIT* (p.Leu678Phe) and *BRAF* (V600E). The presence of these mutations, particularly in the *KIT* gene, suggests a possible contribution to the molecular pathogenesis of PTC [3].

The classification of these VUS variants remains challenging, especially in underrepresented populations such as Latin Americans, where genomic data are scarce in global databases like ClinVar and gnomAD. This underrepresentation leads to a higher prevalence of VUS and complicates variant interpretation [34]. Therefore, accurate clinical interpretation is vital for the eventual reclassification of VUS as either benign or pathogenic [35].

In addition to variant classification, various studies have shown that ancestral background can influence DNA methylation patterns, gene expression levels, and variant frequencies, all of which may affect cancer susceptibility, cancer development, and treatment responses [44]. The ancestral composition analysis of the women in this study revealed a majority Native American component (62.4%), followed by European (31.3%) and African (6.3%) ancestry. The identification of genomic variants, along with significant disparities in risk allele frequencies across populations, supports the development of population-specific genetic panels and the adoption of genotype-guided prescribing practices [45]. Nevertheless, a major challenge remains: the limited genetic diversity represented in clinical and research studies, which have historically prioritized individuals of European ancestry. Although recent efforts have improved the inclusion of underrepresented groups, broader representation is still urgently needed to ensure that the benefits of precision medicine are distributed equitably and applied on a global scale [46].

Thus, liquid biopsy represents a minimally invasive approach capable of providing valuable genomic insights in admixed populations, particularly in the context of thyroid cancer. This study encountered several challenges, notably the low concentrations of ctDNA extracted from plasma samples of Ecuadorian women. These low yields may be attributed to the extraction method employed or to the early stage of disease, which is typically associated with reduced ctDNA levels [47]. Nevertheless, the high concordance of genomic variants between paired samples (81.69%) indicates consistent detection across the cohort, mainly for benign variants and VUSs. On the other hand, the detection of pathogenic variants in ctDNA was notably inconsistent compared to those identified in tumor tissue, particularly with respect to *BRAF* gene mutations.

## 4. Materials and Methods

### 4.1. Inclusion and Exclusion Criteria

The present study included 36 Ecuadorian female patients diagnosed with PTC who underwent partial or total thyroidectomy. Subjects who had undergone prior cancer therapy, or subjects who declined participation, and male individuals were excluded.

### 4.2. Collection of Blood and Tumor Tissue

Peripheral blood samples were collected in EDTA tubes prior the surgical procedure, following the signing of the informed consent. Tumor tissue samples were obtained during thyroidectomies from regions suspected of malignancy.

### 4.3. ctDNA and Tumor DNA Extraction

To maintain sample integrity, the plasma from all samples was separated within 2 h of blood collection. Plasma was initially separated by centrifugation at 1900× *g* for 10 min at 4 °C. A second centrifugation at 16,000× *g* for 10 min was performed to eliminate any residual cellular debris. In cases where immediate processing was not feasible, samples were stored at −80 °C to preserve plasma quality. The initial plasma input ranged from 3 to 4 mL. ctDNA extraction was performed using the QIAamp MinElute cfDNA Mini Kit (Qiagen, Hilden, Germany), according to the manufacturer’s protocol [48,49]. For tumor DNA extraction, the PureLink Genomic DNA mini kit (Thermo Fisher, Waltham, MA, USA) was utilized, following the manufacturer’s protocol, which is optimized for this type of samples [50].

### 4.4. Next-Generation Sequencing (NGS)

Next-Generation Sequencing was performed on both tumor tissue and ctDNA samples using the TruSight Tumor 15 (Illumina, San Diego, CA, USA), which targets 15 different cancer-associated genes, according to the Illumina protocol [51]. This panel includes genes with well-established roles in tumorigenesis, including those relevant to thyroid cancer. Somatic variant calling was conducted using the Trusigh Tumor 15 software 2.0.1 analysis platform by Illumina, which utilizes a banded Smith Waterman algorithm for read alignment using the UCSC hg19 reference genome as well as the Illumina Somatic Variant Caller software 5.2.9.22.

### 4.5. Genetic Variants’ Assessment

Following variant calling, all identified variants were subjected to a multi-step filtering process to ensure high-confidence results. Initial quality control filtering included the assessment of variant-level quality metrics such as the Q-score, coverage, strand bias, and variant allele. For insertions and deletions (InDels), additional filtering was applied to exclude those located within low-complexity or reference repeat regions, which are prone to mapping errors.

Subsequently, variants were filtered based on their functional classification. Synonymous variants, non-coding variants, such as those in untranslated regions, upstream/downstream regions, and intronic variants not located within canonical splice sites were excluded from downstream analyses. This step was performed to prioritize cancer-associated variants, including missense, nonsense, frameshift, canonical splice site, and other exonic or regulatory variants. Variants that failed to meet any of the quality criteria described were excluded from further interpretation and reporting.

The pathogenicity of the variants was determined using the Franklin variant interpretation platform (Genoox, Tel Aviv, Israel) and was based on the guidelines of the American College of Medical Genetics and Genomics (ACMG), which included analyses of computational evidence, patient’s phenotype or family history, population data, in silico modeling, functional data, and frequency of the variants.

### 4.6. Genetic Ancestry Analyses

**Figure 3:**
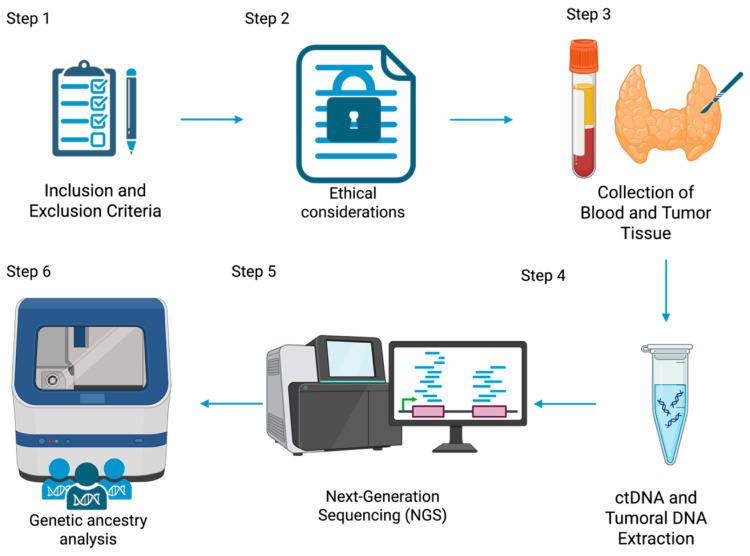
Overview of the experimental workflow for the genomic analysis of thyroid cancer using liquid biopsy and tumor tissue. The diagram illustrates the main steps involved in the study, including data collection and informed consent signing, ethical considerations, blood and tumor tissue samples collection, DNA extraction, next-generation sequencing (NGS), and ancestry determination. Created in https://BioRender.com.

To determine the ancestral background of each participant, tumor DNA was genotyped using Ancestry Informative Markers (AIMs), consisting of InDel polymorphisms. The protocol published by Zambrano et al. (2019) [52] was followed. Fragment analysis was performed via capillary electrophoresis using a 3500 Genetic Analyzer (Applied Biosystems, Waltham, MA, USA). Figure 3 represents the experimental workflow of the present study.

### 4.7. Ethical Considerations

The study protocol was reviewed and approved by the Ethics Committee (Comité de Ética en Investigación en Seres Humanos, CEISH) from Universidad Internacional SEK (code: DIS-CEISH-17-UISEK-21). All participants provided written informed consent prior to inclusion.

## 5. Conclusions

This study underscores the potential of liquid biopsy as a complementary, minimally invasive diagnostic tool for PTC. A high degree of concordance was observed between genetic variants detected in tumor tissue and ctDNA, particularly in key thyroid cancer genes such as the *TP53* gene. However, some variants were exclusive to either tumor tissue or ctDNA, reflecting the genetic heterogeneity of each sample type. Additionally, the identification of VUSs, such as in the *KIT* gene and benign variants in genes like *TP53*, highlights the critical need for accurate variant classification, especially in underrepresented populations.

Future research should focus on expanding genomic reference databases to enhance the representation of Latin American populations. Additionally, standardizing and optimizing protocols for the extraction and analysis of ctDNA, along with the clinical validation of variant interpretation, will be critical. These efforts are fundamental in improving the accuracy of variant classification and in promoting equitable access to precision oncology for underrepresented populations.

## Data Availability

Due to the size of the information, data may be made available upon considerable request.

## Supplementary Materials

The following supporting information can be downloaded at: https://www.mdpi.com/article/10.3390/ijms26146987/s1.
