# Supplementary material for: Evaluating Liquid Biopsy for Circulating Tumor DNA (ctDNA) Detection as a Complementary Diagnostic Tool in Thyroid Cancer Among Ecuadorian Women
Source: Int J Mol Sci. 2025 Jul 21;26(14):6987. doi: 10.3390/ijms26146987 (PMC12295895; doi:10.3390/ijms26146987)
Supplement: Supplementary file 1 [file ijms-26-06987-s001.zip › ijms-3722741-supplementary.pdf]

Supplementary Table S1. Detailed description of each cancer-associated variant.

Patient ID	Age	Gene	HGVSP DNA ref	HGVS protein ref	Consequence	Genotype	ACMG Guidelines	Evidence	Suggested classification
260cf-T	40	*MET*	NM_000245.4:c.3836G>A	NP_000236.2:p.Arg1279Lys	Missense Variant	Heterozygous	PM2_moderate	Population data	VUS
		TP53	NM_000546.6:c.793C>A	ENSP00000269305.4:p.Leu265Met	Missense Variant	Heterozygous	BS3_supporting	Functional studies	Likely pathogenic
							PM2_moderate	Population data	Likely pathogenic
							PM5_moderate	Effect on Protein	Likely pathogenic
							PM1_moderate	Functional data	Likely pathogenic
							PP3_supporting	In-silico prediction	Likely pathogenic
265cf-T	57	*TP53*	NM_000546.6:c.215C>G	NP_000537.3:p.Pro72Arg	Missense Variant	Heterozygous	BA1_very strong	Population data	Benign
							BS2_strong	Population data	Benign
							BP6_supporting	Reputable source data	Benign
		ERBB2	NM_004448.4:c.1963A>G	NP_004439.2:p.Ile655Val	Missense Variant	Heterozygous	PP2_supporting	Functional data	Benign
							BA1_very strong	Population data	Benign
							BS2_strong	Population data	Benign
							BP6_strong	Reputable source data	Benign
265T	57	*PIK3CA*	NM_006218.4:c.1571G>A	NP_006209.2:p.Arg524Lys	Missense Variant	Heterozygous	PM2_moderate	Population data	VUS
							PP2_supporting	Functional data	VUS
		BRAF	NM_004333.6:c.1799T>A	NP_004324.2:p.Val600Glu	Missense Variant	Heterozygous	PS4_strong	Case control studies	Pathogenic
							PM1_moderate	Functional data	Pathogenic
							PP2_supporting	Functional data	Pathogenic
							PM2_moderate	Population data	Pathogenic
							PM5_moderate	Effect on Protein	Pathogenic
							PP3_supporting	In-silico prediction	Pathogenic
							PP5_no influence	Reputable source data	Pathogenic
267cf-T	61	*KIT*	NM_000222.3:c.1621A>C	NP_000213.1:p.Met541Leu	Missense Variant	Heterozygous	BA1_very strong	Population data	Benign
							BS2_strong	Population data	Benign
							BP6_supporting	Reputable source data	Benign
		TP53	NM_000546.6(TP53):c.215C>G	NP_000537.3:p.Pro72Arg	Missense Variant	Heterozygous	BA1_very strong	Population data	Benign

							BS2_strong	Population data	Benign
							BP6_supporting	Reputable source data	Benign
267T		*BRAF*	NM_004333.6:c.1799T>A	NP_004324.2:p.Val600Glu	Missense Variant	Heterozygous	PS4_strong	Case control studies	Pathogenic
							PM1_moderate	Functional data	Pathogenic
							PP2_supporting	Functional data	Pathogenic
							PM2_moderate	Population data	Pathogenic
							PM5_moderate	Effect on Protein	Pathogenic
							PP3_supporting	In-silico prediction	Pathogenic
							PP5_no influence	Reputable source data	Pathogenic
268cf-T	60	*AKT1*	NM_005163.2:c.61A>C	NP_005154.2:p.Thr21Pro	Missense Variant	Heterozygous	PM2_moderate	Population data	VUS
							PP2_supporting	Functional data	VUS
		DHRS7B	NM_015510.5:c.83T>G	NP_056325.2:p.Leu28Arg	Missense Variant	Heterozygous	PM2_moderate	Population data	VUS
							PP3_moderate	In-silico prediction	VUS
			NM_015510.5 c.101T>G	NP_001317088.1:p.Val34Gly	Missense Variant	Heterozygous	PM2_moderate	Population data	VUS
			NM_015510.5:c.105C>G	NP_056325.2:p.Phe35Leu	Missense Variant	Heterozygous	PM2_moderate	Population data	VUS
			NM_015510.5:c.110T>C	p.Leu37Pro	Missense Variant	Heterozygous	PM2_moderate	Population data	VUS
							PP3_moderate	In-silico prediction	VUS
			NM_015510.5:c.110T>G	p.Leu37Arg	Missense Variant	Heterozygous	PM2_moderate	Population data	VUS
							PP3_moderate	In-silico prediction	VUS
		EGFR	NM_005228.5:c.2113A>G	NP_005219.2:p.Arg705Gly	Missense Variant	Heterozygous	PM2_moderate	Population data	VUS
							PP3_supporting	In-silico prediction	VUS
			NM_005228.5:c.2177T>G	NP_005219.2:p.Val726Gly	Missense Variant	Heterozygous	PM2_moderate	Population data	VUS
							PP3_moderate	In-silico prediction	VUS
			NM_005228.5:c.2112G>T	NP_005219.2:p.Leu704Phe	Missense Variant	Heterozygous	PM2_moderate	Population data	VUS
		ERBB2	NM_004448.4:c.1997T>G	NP_004439.2:p.Val666Gly	Missense Variant	Heterozygous	PM2_moderate	Population data	VUS
							PP2_supporting	Functional data	VUS
			NM_004448.4:c.2960T>C	NP_004439.2:p.Val987Ala	Missense Variant	Heterozygous	PM2_moderate	Population data	VUS
							PP2_supporting	Functional data	VUS
			NM_004448.4:c.2958T>G	NP_001276866.1:p.Phe986Leu	Missense Variant	Heterozygous	PM2_moderate	Population data	VUS
		GUSB	NM_000181.4:1277A>C	p.His426Pro	Missense Variant	Heterozygous	PM2_moderate	Population data	VUS

					PP3_moderate	In-silico prediction	VUS
					PP2_supporting	Functional data	VUS
	NM_000181.4:c.1274A>C	NP_000172.2:p.His425Pro	Missense Variant	Heterozygous	PM2_moderate	Population data	VUS
					PP3_supporting	In-silico prediction	VUS
					PP2_supporting	Functional data	VUS
KIT	NM_000222.3:c.2408G>T	NP_000213.1:p.Gly803Val	Missense Variant	Heterozygous	PM2_moderate	Population data	VUS
					PP3_supporting	In-silico prediction	VUS
					PM1_supporting	Functional data	VUS
NRAS	NM_002524.5:c.384dupA	NP_002515.1:p.Gln129ThrfsTer15	Frameshift variant	Heterozygous	PM2_moderate	Population data	VUS
	NM_002524.5:c.212A>C	NP_002515.1:p.Tyr71Ser	Missense Variant	Heterozygous	PM2_moderate	Population data	VUS
					PM1_supporting	Functional data	VUS
					PP2_supporting	Functional data	VUS
					PP3_supporting	In-silico prediction	VUS
	NM_002524.5:c.-17-2A>C	n/a	Splice Acceptor Variant	Heterozygous			
	NM_002524.5:c.386A>C	NP_002515.1:p.Gln129Pro	Missense Variant	Heterozygous	PM2_moderate	Population data	VUS
					PP3_moderate	In-silico prediction	VUS
					PP2_supporting	Functional data	VUS
TP53	NM_000546.6:c.776A>C	NP_000537.3:p.Asp259Ala	Missense Variant	Heterozygous	PS4_moderate	Case control studies	Likely Pathogenic
					PM2_moderate	Population data	Likely Pathogenic
					PM5_moderate	Effect on Protein	Likely Pathogenic
					PP3_supporting	In-silico prediction	Likely Pathogenic
	NM_000546.6:c.455dupC	NP_000537.3:p.Pro153AlafsTer28	Frameshift variant	Heterozygous	PS4_moderate	Case control studies	Pathogenic
					PVS1_strong	Effect on Protein	Pathogenic
					PM2_moderate	Population data	Pathogenic
	NM_000546.6:c.215C>G	NP_000537.3:p.Pro72Arg	Missense Variant	Heterozygous	BA1_very strong	Population data	Benign
					BS2_strong	Population data	Benign
					BP6_supporting	Reputable source data	Benign
	NM_000546.6:c.220dupG	NP_000537.3:p.Ala74GlyfsTer75	Frameshift variant	Heterozygous	PVS1_very strong	Effect on Protein	Likely Pathogenic
					PM2_moderate	Population data	Likely Pathogenic
	NM_000546.6:c.219_220dupGG	NP_000537.3:p.Ala74GlyfsTer50	Frameshift variant	Heterozygous	PVS1_very strong	Effect on Protein	Likely Pathogenic

							PM2_moderate	Population data	Likely Pathogenic
271T	40	*BRAF*	NM_004333.6:c.1799T>G	NP_004324.2:p.Val600Glu	Missense Variant	Heterozygous	PS4_strong	Case control studies	Pathogenic
							PM1_moderate	Functional data	Pathogenic
							PP2_supporting	Functional data	Pathogenic
							PM2_moderate	Population data	Pathogenic
							PM5_moderate	Effect on Protein	Pathogenic
							PP3_supporting	In-silico prediction	Pathogenic
							PP5_no influence	Reputable source data	Pathogenic
272cf-T	40	*TP53*	NM_000546.6(TP53):c.215C>G	NP_000537.3:p.Pro72Arg	Missense Variant	Heterozygous	BA1_very strong	Population data	Benign
							BS2_strong	Population data	Benign
							BP6_supporting	Reputable source data	Benign
273T	53	*BRAF*	NM_004333.6:c.1799T>G	NP_004324.2:p.Val600Glu	Missense Variant	Heterozygous	PS4_strong	Case control studies	Pathogenic
							PM1_moderate	Functional data	Pathogenic
							PP2_supporting	Functional data	Pathogenic
							PM2_moderate	Population data	Pathogenic
							PM5_moderate	Effect on Protein	Pathogenic
							PP3_supporting	In-silico prediction	Pathogenic
							PP5_no influence	Reputable source data	Pathogenic
274cf-T	49	*TP53*	NM_000546.6:c.215C>G	NP_000537.3:p.Pro72Arg	Missense Variant	Homozygous	BA1_very strong	Population data	Benign
							BS2_strong	Population data	Benign
							BP6_supporting	Reputable source data	Benign
274T	49	*BRAF*	NM_004333.6:c.1799T>G	NP_004324.2:p.Val600Glu	Missense Variant	Heterozygous	PS4_strong	Case control studies	Pathogenic
							PM1_moderate	Functional data	Pathogenic
							PP2_supporting	Functional data	Pathogenic
							PM2_moderate	Population data	Pathogenic
							PM5_moderate	Effect on Protein	Pathogenic
							PP3_supporting	In-silico prediction	Pathogenic
							PP5_no influence	Reputable source data	Pathogenic

276cf-T	39	*TP53*	NM_000546.6(TP53):c.215C>G	NP_000537.3:p.Pro72Arg	Missense Variant	Heterozygous	BA1_very strong	Population data	Benign
							BS2_strong	Population data	Benign
							BP6_supporting	Reputable source data	Benign
276T	39	*BRAF*	NM_004333.6:c.1799T>A	NP_004324.2:p.Val600Glu	Missense Variant	Heterozygous	PS4_strong	Case control studies	Pathogenic
							PM1_moderate	Functional data	Pathogenic
							PP2_supporting	Functional data	Pathogenic
							PM2_moderate	Population data	Pathogenic
							PM5_moderate	Effect on Protein	Pathogenic
							PP3_supporting	In-silico prediction	Pathogenic
							PP5_no influence	Reputable source data	Pathogenic
278cf-T	44	*TP53*	NM_000546.6:c.215C>G	NP_000537.3:p.Pro72Arg	Missense Variant	Homozygous	BA1_very strong	Population data	Benign
							BS2_strong	Population data	Benign
							BP6_supporting	Reputable source data	Benign
279cf-T	68	*TP53*	NM_000546.6:c.215C>G	NP_000537.3:p.Pro72Arg	Missense Variant	Homozygous	BA1_very strong	Population data	Benign
							BS2_strong	Population data	Benign
							BP6_supporting	Reputable source data	Benign
		GUSB	NM_000181.4:c.1946T>C	NP_000172.2:p.Leu649Pro	Missense Variant	Heterozygous	PP2_supporting	Functional data	Benign
							BA1_very strong	Population data	Benign
							BS2_strong	Population data	Benign
							BP6_strong	Reputable source data	Benign
281cf-T		*TP53*	NM_000546.6(TP53):c.215C>G	NP_000537.3:p.Pro72Arg	Missense Variant	Homozygous	BA1_very strong	Population data	Benign
							BS2_strong	Population data	Benign
							BP6_supporting	Reputable source data	Benign
286T	61	*BRAF*	NM_004333.6:c.1799T>G	NP_004324.2:p.Val600Glu	Missense Variant	Heterozygous	PS4_strong	Case control studies	Pathogenic
							PM1_moderate	Functional data	Pathogenic
							PP2_supporting	Functional data	Pathogenic
							PM2_moderate	Population data	Pathogenic
							PM5_moderate	Effect on Protein	Pathogenic

							PP3_supporting	In-silico prediction	Pathogenic
							PP5_no influence	Reputable source data	Pathogenic
293cf-T	42	*TP53*	NM_000546.6:c.215C>G	NP_000537.3:p.Pro72Arg	Missense Variant	Heterozygous	BA1_very strong	Population data	Benign
							BS2_strong	Population data	Benign
							BP6_supporting	Reputable source data	Benign
		ERBB2	NM_004448.4:c.1963A>G	NP_004439.2:p.Ile655Val	Missense Variant	Heterozygous	PP2_supporting	Functional data	Benign
							BA1_very strong	Population data	Benign
							BS2_strong	Population data	Benign
							BP6_strong	Reputable source data	Benign
294T	61	*BRAF*	NM_004333.6:c.1799T>A	NP_004324.2:p.Val600Glu	Missense Variant	Heterozygous	PS4_strong	Case control studies	Pathogenic
							PM1_moderate	Functional data	Pathogenic
							PP2_supporting	Functional data	Pathogenic
							PM2_moderate	Population data	Pathogenic
							PM5_moderate	Effect on Protein	Pathogenic
							PP3_supporting	In-silico prediction	Pathogenic
							PP5_no influence	Reputable source data	Pathogenic
295cf-T	54	*ERBB2*	NM_004448.4:c.1963A>G	NP_004439.2:p.Ile655Val	Missense Variant	Heterozygous	PP2_supporting	Functional data	Benign
							BA1_very strong	Population data	Benign
							BS2_strong	Population data	Benign
							BP6_strong	Reputable source data	Benign
		TP53	NM_000546.6:c.215C>G	NP_000537.3:p.Pro72Arg	Missense Variant	Homozygous	BA1_very strong	Population data	Benign
							BS2_strong	Population data	Benign
							BP6_supporting	Reputable source data	Benign
296cf-T	62	*TP53*	NM_000546.6:c.215C>G	NP_000537.3:p.Pro72Arg	Missense Variant	Homozygous	BA1_very strong	Population data	Benign
							BS2_strong	Population data	Benign
							BP6_supporting	Reputable source data	Benign
		GUSB	NM_000181.4:c.1946T>C	NP_000172.2:p.Leu649Pro	Missense Variant	Homozygous	PP2_supporting	Functional data	Benign
							BA1_very strong	Population data	Benign

							BS2_strong	Population data	Benign
							BP6_strong	Reputable source data	Benign
297cf-T	42	*TP53*	NM_000546.6(TP53):c.215C>G	NP_000537.3:p.Pro72Arg	Missense Variant	Homozygous	BA1_very strong	Population data	Benign
							BS2_strong	Population data	Benign
							BP6_supporting	Reputable source data	Benign
297T	42	*BRAF*	NM_004333.6:c.1799T>A	NP_004324.2:p.Val600Glu	Missense Variant	Heterozygous	PS4_strong	Case control studies	Pathogenic
							PM1_moderate	Functional data	Pathogenic
							PP2_supporting	Functional data	Pathogenic
							PM2_moderate	Population data	Pathogenic
							PM5_moderate	Effect on Protein	Pathogenic
							PP3_supporting	In-silico prediction	Pathogenic
							PP5_no influence	Reputable source data	Pathogenic
299cf-T	84	*BRAF*	NM_004333.6:c.1799T>G	NP_004324.2:p.Val600Glu	Missense Variant	Heterozygous	PS4_strong	Case control studies	Pathogenic
							PM1_moderate	Functional data	Pathogenic
							PP2_supporting	Functional data	Pathogenic
							PM2_moderate	Population data	Pathogenic
							PM5_moderate	Effect on Protein	Pathogenic
							PP3_supporting	In-silico prediction	Pathogenic
							PP5_no influence	Reputable source data	Pathogenic
		ERBB2	NM_004448.4:c.3214A>G	NP_004439.2:p.Arg1072Gly	Missense Variant	Heterozygous	PM2_moderate	Population data	VUS
							PP2_supporting	Functional data	VUS
							BP4_supporting	In-silico prediction	VUS
300cf-T		*TP53*	NM_000546.6:c.215C>G	NP_000537.3:p.Pro72Arg	Missense Variant	Heterozygous	BA1_very strong	Population data	Benign
							BS2_strong	Population data	Benign
							BP6_supporting	Reputable source data	Benign
		ERBB2	NM_004448.4:c.1963A>G	NP_004439.2:p.Ile655Val	Missense Variant	Heterozygous	PP2_supporting	Functional data	Benign
							BA1_very strong	Population data	Benign
							BS2_strong	Population data	Benign

							BP6_strong	Reputable source data	Benign
302cf-T	48	*TP53*	NM_000546.6(TP53):c.215C>G	NP_000537.3:p.Pro72Arg	Missense Variant	Heterozygous	BA1_very strong	Population data	Benign
							BS2_strong	Population data	Benign
							BP6_supporting	Reputable source data	Benign
303cf-T	33	*No variants detected*							
305cf-T	37	*TP53*	NM_000546.6:c.215C>G	NP_000537.3:p.Pro72Arg	Missense Variant	Homozygous	BA1_very strong	Population data	Benign
							BS2_strong	Population data	Benign
							BP6_supporting	Reputable source data	Benign
306cf-T	46	*TP53*	NM_000546.6:c.215C>G	NP_000537.3:p.Pro72Arg	Missense Variant	Homozygous	BA1_very strong	Population data	Benign
							BS2_strong	Population data	Benign
							BP6_supporting	Reputable source data	Benign
308cf-T	50	*No variants detected*							
312cf-T	57	*No variants detected*							
313cf-T	62	*No variants detected*							
320cf-T	75	*No variants detected*							
321cf-T	32	*KIT*	NM_000222.3:c.1444G>A	NP_000213.1:p.Ala482Thr	Missense Variant	Heterozygous	PM2_moderate	Population data	VUS
							BP4_supporting	In-silico prediction	VUS
322cf-T	66	*DHRS7B*	NM_001330159.3:c.79C>T	NP_001380586.1:p.Gln42Ter	stop_gained	Heterozygous	PM2_moderate	Population data	VUS
		DHRS7B	NM_015510.5:c.923T>A	NP_001317088.1:p.Phe308Tyr	Missense Variant	Heterozygous	PM2_moderate	Population data	VUS
		ERBB2	NM_004448.4:c.1963A>G	NP_004439.2:p.Ile655Val	Missense Variant	Heterozygous	PP2_supporting	Functional data	Benign
							BA1_very strong	Population data	Benign
							BS2_strong	Population data	Benign
							BP6_strong	Reputable source data	Benign
			NM_004448.4:c.2546G>A	NP_004439.2:p.Arg849Gln	Missense Variant	Heterozygous	PM2_moderate	Population data	VUS
							PP3_moderate	In-silico prediction	VUS
							PP2_supporting	Functional data	VUS
		KIT	NM_000222.3:c.1444G>A	NP_000213.1:p.Ala482Thr	Missense Variant	Heterozygous	PM2_moderate	Population data	VUS
							BP4_supporting	In-silico prediction	VUS
		PIK3CA	NM_006218.4:c.1604C>T	NP_006209.2:p.Ser535Phe	Missense Variant	Heterozygous	PM2_moderate	Population data	VUS
							PM1_moderate	Functional data	VUS
							PP2_supporting	Functional data	VUS
		TP53	NM_000546.6:c.215C>G	NP_000537.3:p.Pro72Arg	Missense Variant	Heterozygous	BA1_very strong	Population data	Benign

							BS2_strong	Population data	Benign
							BP6_supporting	Reputable source data	Benign
324cf-T	54	*KIT*	NM_000222.3:c.1444G>A	NP_000213.1:p.Ala482Thr	Missense Variant	Heterozygous	PM2_moderate	Population data	VUS
							BP4_supporting	In-silico prediction	VUS
		ERBB2	NM_004448.4:c.2546G>A	NP_004439.2:p.Arg849Gln	Missense Variant	Heterozygous	PM2_moderate	Population data	VUS
							PP3_moderate	In-silico prediction	VUS
							PP2_supporting	Functional data	VUS
325cf-T	37	*TP53*	NM_000546.6:c.1081G>A	NP_000537.3:p.Gly361Arg	Missense Variant	Heterozygous	PM2_moderate	Population data	VUS
		KIT	NM_000222.3:c.1444G>C	NP_000213.1:p.Ala482Pro	Missense Variant	Heterozygous	PM2_moderate	Population data	VUS
							BP4_supporting	In-silico prediction	VUS
		ERBB2	NM_004448.4:c.2546G>A	NP_001276866.1:p.Arg849Gln	Missense Variant	Heterozygous	PM2_moderate	Population data	VUS
							PP3_moderate	In-silico prediction	VUS
							PP2_supporting	Functional data	VUS
325T		*BRAF*	NM_004333.6:c.1799T>A	NP_004324.2:p.Val600Glu	Missense Variant	Heterozygous	PS4_strong	Case control studies	Pathogenic
							PM1_moderate	Functional data	Pathogenic
							PP2_supporting	Functional data	Pathogenic
							PM2_moderate	Population data	Pathogenic
							PM5_moderate	Effect on Protein	Pathogenic
							PP3_supporting	In-silico prediction	Pathogenic
							PP5_no influence	Reputable source data	Pathogenic
325cf		*ERBB2*	NM_004448.4:c.2546G>A	NP_004439.2:p.Arg849Gln	Missense Variant	Heterozygous	PM2_moderate	Population data	VUS
							PP3_moderate	In-silico prediction	VUS
							PP2_supporting	Functional data	VUS
326cf	66	*BRAF*	NM_004333.6:c.1799T>A	NP_004324.2:p.Val600Glu	Missense Variant	Heterozygous	PS4_strong	Case control studies	Pathogenic
							PM1_moderate	Functional data	Pathogenic
							PP2_supporting	Functional data	Pathogenic
							PM2_moderate	Population data	Pathogenic
							PM5_moderate	Effect on Protein	Pathogenic
							PP3_supporting	In-silico prediction	Pathogenic

							PP5_no influence	Reputable source data	Pathogenic
326cf-T		*TP53*	NM_000546.6(TP53):c.215C>G	NP_000537.3:p.Pro72Arg	Missense Variant	Heterozygous	BA1_very strong	Population data	Benign
							BS2_strong	Population data	Benign
							BP6_supporting	Reputable source data	Benign
327cf-T	52	*TP53*	NM_000546.6:c.215C>G	NP_000537.3:p.Pro72Arg	Missense Variant	Homozygous	BA1_very strong	Population data	Benign
							BS2_strong	Population data	Benign
							BP6_supporting	Reputable source data	Benign
328cf-T	64	*TP53*	NM_000546.6:c.215C>G	NP_000537.3:p.Pro72Arg	Missense Variant	Heterozygous	BA1_very strong	Population data	Benign
							BS2_strong	Population data	Benign
							BP6_supporting	Reputable source data	Benign
		GUSB	NM_000181.4:c.1946T>C	NP_000172.2:p.Leu649Pro	Missense Variant	Heterozygous	PP2_supporting	Functional data	Benign
							BA1_very strong	Population data	Benign
							BS2_strong	Population data	Benign
							BP6_strong	Reputable source data	Benign
		ERBB2	NM_004448.4:c.1963A>G	NP_004439.2:p.Ile655Val	Missense Variant	Heterozygous	PP2_supporting	Functional data	Benign
							BA1_very strong	Population data	Benign
							BS2_strong	Population data	Benign
							BP6_strong	Reputable source data	Benign
329cf-T	59	*TP53*	NM_000546.6:c.215C>G	NP_000537.3:p.Pro72Arg	Missense Variant	Homozygous	BA1_very strong	Population data	Benign
							BS2_strong	Population data	Benign
							BP6_supporting	Reputable source data	Benign
